# Juvenile Homicide Offenders: Factors in Desistance after Incarceration

**DOI:** 10.3390/ijerph20032354

**Published:** 2023-01-28

**Authors:** Norair Khachatryan, Kathleen M. Heide

**Affiliations:** 1 School of Criminology and Criminal Justice, Northeastern University, Boston, MA 02115, USA; 2 Department of Criminology, University of South Florida, Tampa, FL 33620, USA

**Keywords:** juvenile homicide offenders, recidivism, desistance, qualitative study, post-incarceration adjustment

## Abstract

While several prior studies have examined the prevalence and predictors of recidivism among juvenile homicide offenders (JHOs), much less scholarly attention has been devoted to exploring the post-release factors that influence JHOs to desist from criminal behavior. Given relatively recent rulings by the U.S. Supreme Court, individuals who commit homicide offenses as juveniles are less likely to spend the rest of their lives in prison. Accordingly, it is important to understand the factors associated with desistance in the post-incarceration lives of JHOs. The present study was designed to assess the effects of post-release factors on JHOs’ recidivism outcomes, using a sample of 19 male JHOs from a southeastern U.S. state who were convicted as adults and sentenced to serve time in prison in the 1980s. These men were interviewed approximately 35 years after their original homicide offense about their adjustment to life in prison and after release, as well as their reasons for engaging in criminal behavior during adolescence. Thematic qualitative analysis was used to identify the post-release factors that were prevalent in the lives of the JHOs who desisted from crime. These five factors included avoiding old neighborhood and friends, positive intimate relationship, stable employment, human agency, and generativity. The implications of the findings for the prevention of recidivism among JHOs, as well as avenues for future research, are discussed.

## 1. Introduction

Murder by juvenile offenders (i.e., individuals under the age of 18) has generated a great deal of interest and concern in the United States since the early 1980s. The decade between 1984 and 1993 was characterized by consistent increases in the numbers and rates of arrests for juvenile-perpetrated murders [1]. In 1993, 3284 juveniles were arrested for murder or nonnegligent homicide (hereinafter murder), which represented more than 16% of total arrests for murder in that year, compared to 7% in 1984. The murder rate for adolescents between the ages of 14 and 17, who commit the overwhelming majority of murders by juveniles, in 1993 was 19.3 per 100,000 people, compared to 6.2 per 100,000 people in 1984 [2]. The eruption of youth violence during the aforementioned period was facilitated by an expansion of the drug trade in the country’s urban centers and the widespread availability of firearms [3,4,5]. Various scholars predicted that the high levels of juvenile violence were going to persist into the 21st century [2].

In contrast to the gloomy predictions presented by criminologists and other experts, the rates of arrest for homicide juvenile offenders have decreased overall since 1994 [4,6]. As reported by Heide [6], the percentage of juveniles among offenders arrested for murder decreased by more than half between 1995 and 2014 (15% vs. 7%). In 2019, the last year for which Uniform Crime Report (UCR) data were available, approximately 8% of homicides for which the offender’s age was known were committed by juveniles [7].

Despite the reduction in the rates of juvenile homicide, youths under age 18 continue to commit hundreds of murders every year. Juvenile homicide offenders (hereinafter, JHOs) are also a cause for concern with respect to their propensity for recidivism after release from incarceration. Several prior studies on recidivism among released JHOs have shown that more than 50% of JHOs released from juvenile or adult correctional facilities are rearrested [8,9,10], as further discussed in the literature review. Moreover, many JHOs engage in violence after they are released, and some of them commit new homicide offenses.

### 1.1. Post-Homicide Outcome

Three relatively recent Supreme Court cases have increased the likelihood that juveniles convicted of homicide offenses will be released from incarceration at some point in their lives. In *Roper vs. Simmons* [11], the Supreme Court ruled that imposing the death penalty on offenders who committed murder as juveniles was unconstitutional. The justices determined that juveniles should be held to a lesser degree of culpability, compared to adults, due to a less developed frontal lobe and a higher susceptibility to antisocial influences.

Subsequently, in the *Miller vs. Alabama* [12] case, the Court struck down mandatory life without the possibility of parole sentences for JHOs. In other words, individuals convicted of murder for a crime they committed as juveniles could still be sentenced to life without parole (LWOP), but mitigating factors (e.g., experiencing severe parental maltreatment in childhood) have to be taken into consideration by the judge before JHOs are sentenced.

Lastly, the Court ruled in *Montgomery vs. Louisiana* [13] that the abolition of mandatory LWOP sentences for juvenile offenders applied retroactively, which signified that JHOs who received this sentence prior to 2012 were eligible for resentencing. According to the Marshall Project, hundreds of JHOs have already been resentenced or released from prison since the 2016 ruling [14].

The fact that many future JHOs are likely to serve shorter prison sentences, and many homicide offenders who were supposed to spend the rest of their lives in prison will be released, demonstrate the urgent need to identify the factors that exert the most influence in helping JHOs adjust successfully to society following their release. As discussed in the literature review below, most prior studies on recidivism among JHOs have not examined post-release factors. It is important to gain greater knowledge regarding JHOs’ experiences after they are released from incarceration, and how these experiences contribute to desistance from criminal behavior.

### 1.2. The Present Study

The present study was designed to assess the factors that enable released JHOs to desist from crime, over the course of more than 30 years. Using a sample of 19 JHOs from a single U.S. state, qualitative analyses are employed in this study to identify the most influential post-incarceration factors for desistance. Clearly, pre-incarceration and incarceration-related factors are helpful in understanding why some released JHOs desist from crime and others do not. The identification of post-incarceration factors that reduce the likelihood of recidivism, however, is essential for developing effective intervention programs for JHOs, both during and after incarceration. For example, if employment emerges as an important contributor to desistance among convicted JHOs, the implication is that these offenders need to learn job skills while they are in prison and receive help with obtaining a job after release.

## 2. Literature Review

There has been academic interest in the recidivism of JHOs since the 1970s. Early research in this area focused primarily on parricide offenders (i.e., committed a homicide offense against at least one parent), and typically consisted of small clinical samples. Several of these studies provided promising results regarding the post-homicide reintegration to society of juvenile parricide offenders [15,16,17,18,19]. These studies reported either that the majority of examined juvenile parricide offenders completed a successful transition to society after the homicide or that this group of offenders spent less time in prison than other types of JHOs. Other research on juveniles who killed or attempted to kill their parents presented more mixed results, and highlighted cases of parricide offenders who continued to engage in criminal behavior after their release from custody [20,21,22].

Some prior studies have focused on recidivism outcomes among other subtypes of JHOs. With respect to JHOs who committed sexually oriented homicides, multiple studies have found that the majority of these offenders recidivated after they were released from prison, and their recidivism often included serious violent offenses, including homicide [23,24]. Studies of JHOs comparing homicide circumstances have produced differing results. One study found that JHOs who killed during the commission of another crime (e.g., burglary, robbery) were significantly more likely to recidivate after release than JHOs who killed as a result of an argument or another type of conflict [25]. Conversely, Khachatryan, Heide, and Hummel [26] found no significant differences in recidivism between the two groups of JHOs.

The next section focuses on prior research that has examined recidivism in general samples of convicted JHOs who were released from juvenile or adult correctional institutions, which are the most relevant to the present study. In other words, the studies discussed below did not restrict their analyses to any subtype of JHOs. Subsequently, qualitative research that has explored contributing factors to desistance in samples of formerly incarcerated offenders is discussed, given the focus of the present study.

### 2.1. General Samples of Incarcerated JHOs

To date, 13 studies have analyzed recidivism patterns in moderate to large samples of JHOs who were released from incarceration. In six of these studies, JHOs were released from juvenile correctional facilities [8,10,27,28,29,30]. In the other seven studies, JHOs were released from adult prisons [9,31,32,33,34,35,36]. Eleven of the studies were conducted with U.S. samples, and the remaining two studies were conducted in the Netherlands [10] and Canada [36].

*Study 1.* In a study of JHOs from a midwestern state, Hagan [27] examined a sample of 20 male subjects who were convicted as juveniles of a completed or attempted homicide and released from incarceration in the late 1970s and 1980s. The follow-up period ranged from 5 years to more than 15 years after release. This researcher found that 60% of JHOs (n = 12) had recidivated, and 58% of recidivists (n = 7) had committed a violent act other than murder. The JHOs in the sample, however, were no more likely to recidivate than a control group of 20 non-homicide juvenile offenders.

*Study 2.* Heide and her colleagues [35] followed up on a sample of 59 male JHOs from a southeastern state who were sentenced to adult prison between 1982 and 1984. The sample consisted of offenders who were convicted of murder, attempted murder, or manslaughter. The follow-up period ranged from 1 year to 16 years. The researchers reported that 58% of the JHO released from prison (n = 25) received new prison sentences or were reincarcerated for a parole violation.

*Study 3.* Using the same sample from the study above, Khachatryan and colleagues [9] tracked these 59 JHOs approximately 30 years after their original homicide offense. The authors reported that 88% of the 48 released offenders (n = 42) had been rearrested; 63% of released JHOs (n = 30) had been arrested for a new violent crime. Five of these violent recidivists either killed (n = 4) or attempted to kill (n = 1) again. Logistic regression analyses revealed that JHOs who were incarcerated for six years or less were significantly more likely to be arrested for violence after release from prison than those who were incarcerated for seven years or longer.

*Study 4.* Vries and Liem [10] conducted a follow-up study on a sample of 137 JHOs convicted between 1992 and 2007 in the Netherlands. The follow-up period ranged from 1 year to 16 years. The results indicated that more than half of the sample (59%) was rearrested during the follow-up period. Three percent of all post-release offenses were new completed (n = 2) or attempted (n = 16) homicides. Several demographic and pre-incarceration factors significantly increased the likelihood of recidivism: being male, a lack of self-control, several measures of criminal history (i.e., number of prior offenses, age at first offense, and age at the time of the homicide), and association with delinquent peers. Substance abuse prior to the homicide incident, in contrast to the other variables, was found to decrease the likelihood of recidivism.

*Studies 5, 6, 7, and 8.* Recidivism by JHOs released from juvenile detention facilities managed by the Texas Youth Commission (TYC) was analyzed in four studies by Trulson, Caudill, and their colleagues. In the first study, Trulson and colleagues [29] examined whether juveniles who committed gang-related homicides were more likely to recidivate than other types of JHOs, as well as non-homicide violent offenders. Their sample consisted of 1,804 serious and violent male juvenile offenders, who were released from a juvenile correctional facility between 1987 and 2004. The follow-up period in the study was 3 years.

Multivariate analyses indicated that juvenile gang murderers were significantly more likely to be rearrested for any offense and for a felony offense, compared to the general homicide offenders and non-homicide offenders in the sample. Moreover, general homicide offenders were significantly more likely to be arrested for a new felony offense than non-homicide violent offenders.

In a second TYC study, Caudill and Trulson [8] examined recidivism in a sample of 221 JHOs who were released from TYC facilities between 1987 and 2000. The authors reported that 58% of the sample had been rearrested within the 10 year follow-up period. The risk of recidivism was significantly higher for JHOs who assaulted correctional staff members, served shorter sentences, and accumulated a higher score on a scale that measures behavioral disruption.

In the third TYC study, Trulson and colleagues [30] assessed recidivism in a sample of 238 JHOs released from TYC correctional facilities. Within the five year follow-up period, 58% of JHOs had been rearrested. Offenders who were male and black, as well as those who committed assaults on the ward, were significantly more likely to be rearrested. In contrast, JHOs who participated in an intensive treatment program, served longer sentences, and who participated in fewer assaults against other inmates, were at a significantly lower risk of being rearrested.

In the last TYC study by this group of researchers, Trulson and Caudill [28] tracked 247 JHOs who were sentenced to the TYC in the years 1987–2011. Within the three year follow-up period employed by the authors, 50% of the sample had been rearrested. Offenders who were black, experienced childhood neglect, and assaulted other inmates, were more likely to recidivate after release.

*Study 9.* Recidivism outcomes for 22 JHOs who were released from adult prisons across the state of Massachusetts were examined by DiCataldo and colleagues [31]. The authors employed a mean follow-up period of approximately 8 years. The majority of JHOs in this sample did not recidivate during the study period. The results indicated that 32% of the sample (n = 7) were reconvicted of a crime after release from prison. There were no significant differences between the recidivists and non-recidivists on any of the factors examined.

*Study 10.* In a study of recidivism among juvenile and young adult killers in Canada, McCuish and colleagues [36] analyzed the offending trajectories of 26 young murderers (i.e., killed between the ages of 12 and 19) after release from prison and up to age 28. They also compared the homicide group to young violent (n = 358) and non-violent offenders (n = 139). The researchers reported that 71% of the homicide offenders were reconvicted of a post-release crime, but their crimes tended to be minor and non-violent. No significant differences were found between the homicide group and the other two groups on the likelihood of recidivism and post-release offending trajectories.

*Studies 11, 12, and 13.* The remaining three studies examined reincarceration in the same sample that will be used in the present study. The sample consisted of 20–22 male JHOs who were convicted of homicide offenses in adult court and sentenced to serve time in adult prison in the early 1980s. The follow-up period for these studies was approximately 35 years. In the first study, Heide [32] explored the influence of pre-homicide, incarceration-related, and post-incarceration factors on recommitment to prison for 19 JHOs who had been released. The results indicated that 58% of these men (n = 11) had been reincarcerated during the follow-up period. Logistic regression analyses revealed that JHOs who went back to their old neighborhoods after release from prison were more likely to be reincarcerated, whereas offenders who served longer sentences and those who completed a GED in prison were less likely to be reincarcerated.

In the second study, Heide [33] analyzed data from a series of questions presented to the JHOs about the reasons that they engaged in criminal behavior in the early 1980s. Analyses indicated that JHOs who reported that as kids they lived in neighborhoods in which crime was routine, and those who got involved in crime because the opportunity presented itself, were significantly more likely to be reincarcerated than JHOs who did not report these circumstances.

In a subsequent study that focused on the JHOs’ motivations for engaging in serious criminal behavior before incarceration, Heide [34] found that the psychological (e.g., search for identity) and sociological (e.g., subcultural values) factors identified by the men as influential in their decision to offend were evenly split for the 18 JHOs in the sample. The reasons for criminal involvement did not significantly differentiate between the JHOs who were reincarcerated and those who were not reincarcerated.

### 2.2. Synopsis of the JHO Recidivism Literature

A perusal of the studies reviewed above leads to several important conclusions. First, follow-up studies on JHOs show that large proportions of these offenders recidivate after their release. In fact, most prior studies have found that the majority of them continue engaging in deviant or criminal behavior. Second, longer periods of incarceration are significantly correlated with lower recidivism rates than shorter sentences. Third, a relatively small percentage of JHOs commit new homicide offenses. Fourth, the knowledge regarding the recidivism patterns of different subtypes of JHOs is currently limited. Fifth, prior research has devoted little attention to the effects of post-release factors on recidivism. Lastly, the extant literature on recidivism among JHOs consists primarily of quantitative studies.

### 2.3. Qualitative Research on Offenders Released from Incarceration

Several factors have been identified as important for desistance in prior qualitative studies of former inmates. Laub and Sampson [37] highlighted the role of informal social control in desistance. Specifically, through interviews with a sample of male offenders, they found that stable full-time employment and high levels of attachment toward one’s wife served as turning points away from criminal behavior for these men.

Numerous scholars have examined the contributions of identity transformation and generativity to desistance [38,39,40,41,42]. For example, in his study of formerly incarcerated offenders from England, Maruna [42] found that the desisters in his sample viewed themselves as prosocial individuals and reported a sense of control over their actions. Desisters in several prior qualitative studies have also exhibited generativity, which refers to the desire to help others in society, and young people in particular [43]. The desisters in the study by Aresti, Eatough, and Brooks-Gordon [44], for instance, reported deriving a sense of purpose from working with socially marginalized groups following their release.

Another influential factor in desistance has been found to be social support. Prior qualitative research has demonstrated that support provided to formerly incarcerated offenders by family members or friends [40,45], or the community at large [42], are essential for a successful readjustment to society.

The present study aims to address some of the gaps in the literature mentioned above by examining the factors that are related to JHOs’ desistance from crime. Qualitative analyses are used to explore the post-release experiences of 19 JHOs. This approach allows for a greater understanding of the precise mechanisms that contribute to desistance among the offenders in the sample who ended their involvement in serious criminal behavior following their release for the homicide conviction.

## 3. Methodology

The JHOs in the present study were drawn from a larger sample of 59 juvenile offenders from a southeastern state who were arrested for murder or attempted murder between 1981 and 1983, and who were interviewed in prison between 1983 and 1984 by the second author of the study. The original sample was identified by the Department of Corrections (DOC) in the southeastern state under study. The following inclusion criteria were used: (1) male offender, (2) under the age of 18 at the time of the homicide incident, (3) charged as an adult with murder or attempted murder, (4) convicted as an adult and entered an adult prison between January 1982 and January 1984, (5) incarcerated less than a year at the time of identification by the DOC, and (6) 19 years old or younger at the time of the initial interview.

The sample consisted solely of male offenders due to the fact that JHOs, similar to other types of violent juvenile offenders, have long been predominantly males [46]. The sample contained both murderers and attempted murderers because their homicidal intentions were not found to differ; some of the subjects in the sample did not kill their victim due to such factors as poor marksmanship, the physical stamina of the victim, and the rapid availability of medical care [47,48].

Only JHOs who were processed as adults were included in the sample because the vast majority of juveniles arrested for murder in the early 1980s were treated as adults in the southeastern state from which the sample was selected. For example, close to 90% of juveniles charged with homicide offenses in 1983 were sent to adult court in this state. Lastly, sample subjects had to be incarcerated for less than a year because the researcher sought to interview offenders who were still in their adolescent years and had yet to become institutionalized.

Following the recruitment of sample subjects, in-depth psychosocial interviews were conducted with the 59 JHOs. The research protocol was approved by the Institutional Review Board of the interviewer’s academic institution. Informed consent was obtained from all 59 JHOs prior to beginning the interview. The interviews covered areas such as family history, neighborhood circumstances, school and work history, drug, and alcohol use, dating and sexual history, leisure activities, values and beliefs, history of antisocial behavior, and circumstances behind the original homicide offense. The interviews were supplemented by official records, which included police reports regarding the index homicide offense, pre-homicide delinquent history, family background, education and work history, substance abuse, and court documents. The record data were collected from a variety of sources, such as probation department reports, indictment and charging documents, conviction and sentencing documents, and DOC reports.

### 3.1. Generation of Present Sample

Ten of the 59 JHOs in the original sample were found to be deceased. Two of the offenders died of AIDS before completing their sentence for the murder conviction, one of them died under unknown circumstances after he escaped from prison, one drove a vehicle while intoxicated and was killed in a car accident, one reportedly died from complications related to alcoholism, and another two offenders were murdered; one of them was mistaken for a rival gang member and was stabbed to death, while the other was fatally shot during a robbery. No information on the cause of death was available for the remaining three deceased JHOs.

The follow-up interview protocol was approved by the same Institutional Review Board that approved the original study. The 49 offenders from the larger sample who were found to be alive were contacted by letter, informed of the study’s purpose, and asked to participate in an interview about their experiences in prison and after release. Letters to five of these 49 JHOs were returned and attempts to find a current address were unsuccessful. Twenty-two of the 44 homicide offenders (50%) who were contacted agreed to participate in the study. The response rate in this study is higher than in most studies that examine prison-related issues [49,50]; studies with prisoners that entail sensitive questions typically report a response rate of 25% or less [49].

Chi-square and *t*-test analyses revealed no significant differences on demographic, prior record, and homicide-related variables between the 22 JHOs who agreed to be interviewed and the 22 JHOs who did not participate in the study. The analyses were used to test whether the two groups differed on offender race, growing up in a high-crime neighborhood, pre-homicide arrest record, pre-homicide violent arrest record, the number of pre-homicide arrests, the use of accomplices in the homicide incident, whether the homicide offense victim was a stranger, whether the homicide offense was crime-oriented or conflict-oriented, the type of weapon used in the homicide offense, and time served in prison for the homicide conviction.

The 22 JHOs were interviewed by one of the authors of the study in 2018 and 2019. Eight offenders were interviewed in prison; five of these men had been released and subsequently reincarcerated. Conversely, the remaining 14 offenders were interviewed in several cities across the U.S. Three of the 22 JHOs were excluded from the present study because they had not been released from prison for the index homicide conviction during the 35 year follow-up period. Therefore, the final sample in the present study consists of 19 men who were incarcerated for a homicide offense they committed as juveniles and released from prison at some point.

### 3.2. Follow-Up Interviews

The follow-up interviews were semi-structured and consisted of three main parts: (1) experiences in prison; (2) experiences after first release from incarceration, including any recidivism; and (3) reflections about their involvement in juvenile delinquency and the homicide incident. *The JHOs in the sample were asked to describe their experiences in prison*, with a particular emphasis on how sample subjects fared in prison as adolescents [32]. While discussing incarceration experiences, the following topics were covered:

- Participation in mental health and substance abuse treatment programs;
- Educational attainment;
- Vocational training and work assignment;
- Availability of drugs and alcohol and use of these substances;
- Religious activities;
- Difficulties with other inmates and correctional officers;
- Violent and property victimization experiences;
- Disciplinary misconduct;
- Physical and mental health;
- Overall adjustment to prison conditions;
- Contact with friends and family members;
- Friendship with other inmates;
- Intimate relationships inside and outside prison;
- Plans for the future.

*Regarding the offenders’ lives after their initial release from prison*, the following topics were covered during the interview:

- Places of residence;
- Intimate relationships;
- Employment;
- Educational attainment;
- Participation in treatment programs;
- Post-release supervision;
- Overall views on difficulty of reintegration into society;
- Communication with family members and pre-homicide arrest friends;
- Use of drugs and alcohol;
- Leisure activities;
- Involvement in post-release criminal behavior.

Furthermore, offenders who had been rearrested after their first release from prison were asked to discuss the circumstances behind the arrests, the disposition of the cases, whether or not they had been recommitted to prison, and the effects of reincarceration, if applicable. The analyses presented below focus largely on the JHOs’ lives after they were released.

In the final part of the interview, the offenders were asked to reflect on their participation in criminal behavior in childhood and adolescence, including the index homicide offense. Regarding criminal behavior in general, the JHOs were asked to rate the degree to which 20 distinct theoretically derived factors contributed to their involvement in juvenile crime, on a scale of 1 (= not a factor) to 3 (= a big factor). These factors included peer pressure, being under the influence of drugs and/or alcohol, and living in a neighborhood where crime was common, among others. With respect to the homicide offense, the JHOs were asked to describe how they felt about the incident and whether they thought the crime could have been prevented in any way. The purpose of the former question was to determine whether the subjects felt any remorse for killing or attempting to kill another person. The latter question assessed whether JHOs were aware that they had choices in life, and that they chose to engage in behavior that led to the homicide incident [1,51].

The mean length of the interviews with the 19 JHOs in the final sample was 4.9 h, with a median of 4.5 h. The interview length ranged from 1.5 h to more than 12 h. The interview that surpassed 12 h in length was conducted over the span of two days.

The interviews followed a detailed 40-page protocol. Approximately 100 questions related to the three topic areas described above were prepared by the interviewer in advance. The subjects’ responses typically led to prepared follow-up questions that were tailored to their particular responses. The follow-up interviews were not recorded in order to alleviate any concerns of the subjects regarding disclosure and to encourage candid responses. Instead, subjects’ responses were written down by the interviewer in the 40-page protocol packet, including many direct quotes. Subsequently, the responses were organized by topic area and transcribed. The analyses in this study relied heavily on these transcripts.

### 3.3. Recidivism Data

The decision was made in this study to classify whether a JHO was a desister or not using official criminal records rather than to rely simply on the JHOs’ reports. Official records were employed to determine recidivism in order to account for the possibility that offenders might have omitted certain crimes due to minimization efforts or memory problems. National Criminal Information Center (NCIC) data were collected for all JHOs in an earlier follow-up study that spanned 30 years [9]. These arrest data were supplemented by two data sources for the 35 year follow-up study. Fifteen of the 19 released JHOs remained in the southeastern state where they were arrested for the original homicide offense, and their criminal records were obtained through the central law enforcement agency in that state. These data included information on arrests, dispositions, and incarcerations. Moreover, a background check platform that was determined to be legitimate was utilized to examine the criminal records of the remaining four JHOs, who had relocated to other states after their release from prison.

### 3.4. Plan of Analysis

The analyses in the present study entail two parts. First, various categories of descriptive statistics are presented for the whole sample: demographic characteristics (race, current age, age at the time of the arrest for homicide, whether the JHO grew up in a high-crime neighborhood), childhood maltreatment (abuse, neglect), and prior criminal behavior (childhood abuse, childhood neglect, pre-homicide arrests, pre-homicide arrests for violence), homicide incident and adjudication characteristics (use of accomplices, stranger victim, homicide circumstances, the method of killing, conviction type), and time served in prison for the homicide conviction.

Subsequently, common themes in the lives of the JHOs who desisted after release are explored. Particular attention was devoted in the thematic analysis to factors that have been found to influence desistance in the past, such as marriage, employment, human agency (i.e., an individual’s actions are driven by conscious choices), a change in identity and the development of prosocial values, and a lack of association with antisocial peers [37,38,52]. As shown below, these analyses also rely upon information from the original interviews from the 1980s with the JHOs.

For the purpose of these analyses, a desister was defined as a JHO who met the following criteria: (1) has not been arrested for any serious violent or property offenses, (2) has not been arrested frequently (more than four times) for minor offending, and (3) has not been recommitted to prison for any reason, including violation of parole or probation. Examples of serious violent crimes are index violent crimes from the FBI’s UCR (murder or non-negligent manslaughter, rape, robbery, and aggravated assault), as well as offenses such as simple assault, kidnapping, and firing a weapon. Serious property crimes include several index property crimes from the UCR (burglary, motor vehicle theft, and arson), as well as grand theft (i.e., theft of items worth USD 750 or more) and dealing in stolen property. Frequent minor offending is defined as having been arrested five or more times for misdemeanors and less serious felonies from the date of release from prison for the homicide conviction to the end of the follow-up period, which was November 2020. The five-arrest threshold described above is based on the definition of chronic offending in the study by Wolfgang, Figlio, and Sellin [53].

## 4. Results

The demographic characteristics of the sample, as well as information on the type of homicide conviction the JHOs received, are displayed in Table 1. Close to 70% of JHOs (n = 13) were black, whereas the remaining 32% (n = 6) were white. At the time of the follow-up interview, offenders were between the ages of 50 and 54, with a mean age of 52.5. At the time of their arrest for homicide, the offenders were between the ages of 14 and 17, and their mean age was 15.7. More than 40% (n = 8) of the sample reported spending their childhood in low-income neighborhoods in which violence and visible drug-related transactions were common. Lastly, with respect to homicide conviction, 79% of sample subjects killed their victim; two JHOs (10.5%) were convicted of first-degree murder, 12 JHOs (63%) were convicted of second-degree murder, and a single JHO (5%) was convicted of manslaughter. The remaining four offenders were convicted of attempted murder.

Originally, two thirds of the JHOs in the sample whose victims died were charged with murder in the first degree (10 of 15 JHOs), which made them eligible for the death penalty in the state under study during that period of time. The charge of first-degree murder encompassed murders perpetrated during the commission of a felony (e.g., robbery, burglary), which applied to the majority of sample subjects. Eight of the 10 JHOs who were originally charged with first-degree murder pled guilty to second-degree murder, one of them pled guilty to first-degree murder, and the remaining JHO was convicted of first-degree murder at trial.

Information on the 19 JHOs’ histories of childhood maltreatment and pre-homicide criminal record is displayed in Table 2. There is evidence of childhood abuse for 47% of the JHOs in the sample (n = 8), while more than 70% of the JHOs (n = 14) experienced childhood neglect. The former variable was measured by whether the youth reported experiencing physical or emotional maltreatment, and the latter variable was measured by the degree of parental involvement and monitoring in childhood (e.g., a JHO was neglected if he was able to roam the streets at night without supervision). With respect to criminal history, close to 80% of sample subjects (n = 15) had a criminal record prior to the homicide incident; the highest number of pre-homicide arrests was 16, with a mean of 4.5. More than 40% of them (n = 8) were previously arrested for a violent offense. The violent offenses consisted of robbery, simple assault/battery, and aggravated assault/battery.

The characteristics of the index homicide incidents are provided in Table 3. Approximately 80% of the JHOs in the sample (n = 15) committed the homicide offense with at least one accomplice, and nearly 60% killed a stranger. Close to 75% of JHOs killed or attempted to kill their victims during the commission of another crime, such as a residential burglary or a robbery. Regarding the method of killing, the highest percentage of offenders (42%, n = 8) used a firearm, followed by a knife and a blunt object, both of which were used by 21% of JHOs (n = 4). One JHO in the sample participated in a group killing that involved more than one type of weapon; the victim in this case was struck by a car, hit with a cinder block, and repeatedly kicked in the chest.

Lastly, the offenders in the sample were incarcerated for the homicide conviction with a mean of 159.8 months (approximately 13 years, 4 months), ranging from 25 months (2 years, 1 month) to 422 months (35 years, 2 months). Several JHOs served relatively short sentences because, in the 1980s, prison inmates in the state under study received two days of credit for every day of good behavior. Accordingly, JHOs were often released after serving approximately a third of their sentence. For example, a defendant who was sentenced to serve a life sentence (calculated as 17 years in prison) would have been released after serving 6-7 years, if he/she generally complied with correctional officers’ rules during incarceration. Currently, due to a “truth-in-sentencing” law that was implemented in the mid-1990s in that state and many other states, inmates must serve 85% of their sentence before they become eligible for early release [54].

### 4.1. Thematic Analysis of Desistance

Out of the 19 JHOs in the sample, eight met the criteria for desistance. Since their re-lease from prison for the murder conviction, these offenders have not been rearrested for any serious violent or property offenses, have not been rearrested frequently for minor offending, and have not been reincarcerated in prison for any reason. Six of the desisters have not been rearrested since their release; the two remaining JHOs in this category have accumulated a small number of arrests for minor crimes. One of these JHOs has been rearrested twice, while the other one has been rearrested four times.

Regarding offender characteristics, six of the eight desisters were white and five of them grew up in high-crime neighborhoods. With respect to original homicide incident characteristics, all eight desisters served time in prison for completed homicides; two of them were convicted of first-degree murder and six were convicted of second-degree murder. Half of the desisters committed the original homicide with accomplices. The same number of desisting JHOs killed a victim who was a stranger and committed a crime-oriented killing. Regarding the method of killing, the highest proportion of desisters (n = 3) used a firearm; the remaining five desisters killed their victim using a knife (n = 2), asphyxiation (n = 2), or a blunt object (n = 1).

The interviews conducted with the JHOs reveal five common themes in the lives of JHOs who desisted after their release from prison. The following themes are further explored below: Avoiding old neighborhood and friends, positive intimate relationship, stable employment, human agency, and generativity. Specific statements made by the JHOs are presented while discussing each theme to illustrate the influence of the specific factors on the desistance process. A pseudonym based on the marine alphabet was assigned to each desister to protect his identity.

#### 4.1.1. Avoiding Old Neighborhood and Friends

Six of the eight desisters in the sample did not go back to the neighborhoods where they grew up after they were released from prison, and seven of them avoided spending time with their pre-incarceration friends. For example, Golf grew up in a neighborhood in the northeast with various indicators of disadvantage: many buildings were vacant; illegal drugs were easily obtainable; homeless individuals could be seen drinking alcohol on the street; the sight of sex workers was common; and gangs violently terrorized neighborhood residents. After moving to the southeastern state as a teen, where he was ultimately arrested for homicide, Golf settled in a neighborhood where the sale of illegal drugs was commonplace. Moreover, he frequently consumed drugs with his friends.

Golf was younger than 17 years of age when he was arrested for fatally shooting a man who had previously victimized him, and who was threatening him during the incident. Golf was released from prison after serving approximately five years for the murder and decided not to return to either of the neighborhoods in which he grew up. He felt nostalgic regarding the neighborhood in the northeast where he was born and raised, but decided that returning to that neighborhood “was not a good idea”. After his release, Golf initially remained in the state where he was previously incarcerated, before relocating to a city in the midwest. Furthermore, although he has reconnected with some of his old friends on a social media site, he has not spent time with any pre-incarceration friend since his release from prison. Golf has not been rearrested since he was released from incarceration in the mid-1980s.

Echo and Lima grew up in low-income neighborhoods across the same city in a southeastern state. Their neighborhoods were characterized by violence, gang-related activities, drug trafficking, and the public consumption of alcohol. Furthermore, the JHOs’ friends in adolescence sold drugs and committed assaults. When these individuals were both under the age of 15, they were arrested for the brutal killing of an adult man. Echo was deemed to be the more culpable of the two offenders and spent approximately 25 years in prison. Lima, in contrast, served about seven years. Echo did not return to his old neighborhood or reconnect with his old friends after he was released, including Lima. He initially lived on the street, before settling in the home of a man whom he knew from prison. Subsequently, Echo relocated to a southern state, where his family was living. Echo has not been rearrested since his release from prison more than 10 years ago.

In contrast to Echo, Lima returned to the neighborhood where he grew up shortly after release to live with his parents. Interview data suggest that he eventually rented a home in a different neighborhood, where he lived with a girlfriend and her children at the time of the interview. Similar to Echo, Lima has not reconnected with his pre-incarceration friends since his release and claimed that he “does not want to do so”. Since his release more than 25 years ago, Lima has been arrested twice for relatively minor offenses (DUI and trespassing); he has been crime-free more than 15 years.

#### 4.1.2. Positive Intimate Relationships

The interview data suggest that the intimate partners of three JHOs in the sample took steps to ensure that these men would not resume their involvement in criminal behavior. Shortly after his release from prison, Golf met a woman who worked at a convenience store and reported an “instant connection” with her; she became Golf’s wife after six months of dating. They have been married for more than 30 years and Golf described her as a “good influence” on his life.

Golf’s marriage facilitated his desistance process in several ways. First, Golf and his wife at some point in their marriage relocated to the midwestern city where she grew up, which kept him away from potential negative influences in his home state. Second, Golf joined the military and completed several tours abroad. Following an honorable discharge from the military, Golf experienced health problems and was diagnosed with post-traumatic stress disorder (PTSD), and his wife reportedly provided both instrumental and emotional support in helping him overcome these issues. She contacted a Department of Veterans Affairs (VA) facility in order for Golf to receive medical treatment there. Moreover, she encouraged Golf to participate in individual counseling to alleviate his symptoms of PTSD; he described the counseling he received as a “positive experience” in the interview. Golf’s wife also convinced him to stop his participation in group therapy when she observed that he was becoming an angrier person as a result of these therapy sessions.

Lastly, Golf related that he had a close relationship with his wife’s parents, although they initially opposed his relationship with their daughter. His father-in-law taught him several skills, including roofing, construction, and building decks. At the time of the follow-up interview, Golf reported using these skills while working on a part-time basis in the city where he lived.

Another JHO whose desistance process was facilitated by marriage was Mike. This offender was convicted of second-degree murder after shooting a man to death during an argument over a woman. Mike was under the age of 17 at the time of his arrest and he served approximately 27 years for this conviction. Mike related that after his release from prison, he was completing a work project at a medical facility when he met the woman who eventually became his wife. He described his wife as very supportive of his efforts to lead a successful life after incarceration; for example, she enabled him to receive a bachelor’s degree by paying his tuition, which improved his employment opportunities, as described further below. Moreover, Mike’s wife had adult children from a previous relationship, and raising her grandchildren appeared to have given him a sense of purpose. Mike has not been rearrested since his release from incarceration more than 10 years ago.

Lima has not been married since his release from prison more than 30 years ago. One of the intimate relationships in which he has been involved, however, was beneficial to his desistance process in several ways. He described the girlfriend with whom he was living at the time of the interview as “awesome” and reported that she had compelled him to stop consuming alcohol; this change was an important factor in Lima’s life because, as previously mentioned, he had been rearrested for a DUI since his release from the homicide conviction.

#### 4.1.3. Stable Employment

Most of the desisters in the sample (n = 6) have held stable employment during the follow-up period. Hotel is an example of a JHO who benefited from stability in employment. When he was under the age of 17, Hotel was arrested for fatally shooting another young man during an argument over a hat. He was released from prison after 20 years of incarceration and has not been rearrested for any serious crime since his release. The ability to work appears to be a strong factor in his desistence from serious offending. Hotel’s first job after prison was a full-time security guard. He was working in this position for several years before his employer found out that he was a convicted murderer and fired him. Prior to his dismissal, Hotel reported being held in “high esteem” at this job.

Hotel related that after losing his job as a security guard, he spent several months taking temporary jobs, before deciding to open his own business. According to Hotel, the business entailed providing services such as plumbing, cleaning, removing trash, constructing fences, and remodeling homes. At the time of the follow-up interview, Hotel’s clients included “a lot of realtors and five police officers”. This business enabled him to buy his own home and fully pay for it, which made him very proud of himself.

As discussed above, Hotel has not been arrested for any serious, violent, or property crimes following his release for the homicide conviction, and he has not been reincarcerated. He has been rearrested four times for minor offenses, including petty theft (i.e., he stole items worth less than USD 750), violation of commercial vehicle marking laws, and the possession of ammunition. Hotel stated that he was arrested for possessing ammunition, which is prohibited to him as a convicted felon, because an old bullet was found in a pile of trash in the back of his pickup truck. Hotel explained that he had been “cleaning properties and hauling debris away” within the context of his business, and did not know that the bullet was in the rubble.

The benefits of stable employment could also be seen in Victor’s life. When he was younger than 17 years of age, Victor was arrested for killing a younger family member. He served approximately 16 years in prison for this crime, and initially struggled to obtain a stable job due to his conviction history. A few years after his release, he decided to enter the field of information technology (IT) and was able to turn this profession into a career. He completed a bachelor’s degree and a master’s degree in the field and proceeded to find a job at a large IT company; he worked at that company for more than five years. At the time of the follow-up interview, Victor was employed at a different IT company, where he had been working for approximately five years.

Victor was apprehensive about potentially losing his job one day as a result of his murder conviction; he discussed “always waiting for the other shoe to drop” with respect to the possibility that his criminal record would become known. Nevertheless, Victor described a sense of pride in himself due to his employment-related accomplishments. He has not been rearrested for any offenses since he was released from prison more than 20 years ago.

Some of the desisters discussed previously have also experienced stable employment after they were released from prison. For example, Lima had been towing vehicles on a full-time basis for more than 15 years; he worked at one towing company for more than 10 years and had been working at a different company for approximately five years at the time of the follow-up interview. Moreover, Mike has held several full-time jobs since his release, including working at a restaurant, a security company, and a computer company; he reported “moving up” to higher-level positions at each of these jobs. Mike had been working as an administrator at an institution of higher learning for more than 5 years at the time of the interview. His employment success could be attributed to hard work and his post-release educational attainment; he had completed both associate and bachelor’s degrees.

#### 4.1.4. Human Agency

Interviews with at least four of the desisters displayed evidence of human agency. As described in Section 3, the concept of human agency refers to the conscious choice that some offenders make to reform their lives and desist from criminal behavior [37]. When asked about his feelings at the time of his release from prison, Golf stated: “I made a choice; I am not coming back”. Golf also commented that he was determined to “find a wife and have children”. These statements indicate that Golf made a deliberate decision to desist from crime after he was released and add elements to his life that would facilitate the process of desistance (i.e., marriage and children). Similarly, Mike reported that he made a conscious decision to change the direction of his life. During the first few years of his incarceration, Mike engaged in a great deal of misconduct, including the commission of an aggravated assault against a correctional officer; he stated that he was “playing the fool” during those years. While segregated from the general inmate population due to his disciplinary problems, Mike made a choice to change his behavior and devote effort toward rehabilitation.

As mentioned above, Lima has been arrested twice for minor offenses since his release. He explained his lack of involvement in serious criminal behavior by stating, “I put my priorities first. I have been there and done that”. These statements demonstrate a conscious commitment to avoid serious violations of the law for the purpose of not returning to prison. In the case of Victor, he reported participating in “dozens of interviews” before being hired in the initial period following his release from prison; his perseverance in searching for a legitimate employment opportunity indicates that he was committed to maintaining a prosocial lifestyle, despite the challenges he faced.

#### 4.1.5. Generativity

Evidence of generativity was found in the interviews of at least three desisters. During his interview, Echo described widespread corruption and lawlessness in the prisons in which he served his sentence, including the “torturing of children” by correctional officers and other inmates. Moreover, Echo claimed that many offenders who entered prison as juveniles had been killed because prison administrators were “sticking them with predators”, referring to the lack of protection for juvenile offenders from older, exploitative inmates.

When asked about his purpose in life, Echo stated that he wanted to be “the voice of all the children” who had suffered or died in prison. Although the veracity of his claims regarding the large-scale torture and murder of juvenile prison inmates is questionable, it is evident that his desire to advocate on behalf of incarcerated juvenile offenders has played an important role in his lack of post-release involvement in criminal behavior. The importance of generativity in Echo’s desistance from crime is particularly noteworthy due to the fact that he has struggled with respect to other factors known to influence desistance; for example, he has not held stable employment and was unemployed at the time of the interview. He has also not been involved in any stable intimate relationships since his release.

Another desister whose goal is to help younger people is Oscar. When this offender was under the age of 16, he burglarized an older man’s home along with a friend and killed the victim after he began to scream. Oscar served 30 years in prison for this crime. Following his release, he has sought to encourage juveniles to avoid becoming involved in criminal behavior. Oscar reported speaking about his experiences in prison to a group of high-risk youth between the ages of 5 and 15; he has also visited a school to share his insight regarding crime and incarceration. At his interview, Oscar explained his motivation for helping children by stating: “I want to give back. There is no point if it can’t benefit somebody”. He has not been rearrested since his release from prison more than six years ago.

Indicators of generativity were also found in Romeo’s follow-up interview. When asked about his plans for the future, Romeo commented that his main goal was to “help people”. Moreover, the JHO reported meeting with prison inmates to guide them in preparing for life after incarceration.

The overall applicability of the factors described above to the desisters in the sample is displayed in Table 4. None of the desisters exhibited all six factors throughout their lives after prison. The mean number of desistance factors was 3.6. The JHOs who exhibited the highest number of factors were Golf and Mike (five factors). Notably, the lowest number of factors was exhibited by Hotel (two factors), who was most involved in post-incarceration criminal behavior among the desisters.

## 5. Discussion

The present study was the first to examine the lives of JHOs who had been released from prison up to middle adulthood. The analyses presented above revealed several common experiences in the lives of homicide offenders who had desisted from crime after their release from prison. These findings provide important insight into the factors that most influenced these offenders in ending their involvement in criminal behavior. The factors included the avoidance of pre-incarceration neighborhood and friends, a marriage or intimate relationship with a supportive and prosocial partner, stable employment, a conscious effort to stop engaging in criminal behavior (i.e., human agency), and a desire to help young people avoid the criminal lifestyle and incarceration (i.e., generativity).

The themes of avoiding one’s old neighborhood and pre-incarceration friends were discussed together in Section 4 because they are related to one another: Offenders who settle in the same neighborhood where they lived prior to incarceration are more likely to spend time with their pre-incarceration friends, and consequently resume their involvement in the same type of criminal behavior in which they engaged before they were arrested for the homicide offense [55]. Conversely, JHOs who settle in other neighborhoods following their release are less likely to reconnect with their old criminal friends and may instead form friendships with prosocial individuals. Other important criminogenic factors related to the disadvantaged neighborhoods to which JHOs often return after incarceration are few opportunities for stable employment, the widespread availability of illegal drugs, and a conflict-prone environment due to a higher concentration of individuals who have committed violent offenses [55,56,57,58].

The findings in this study provide evidence that the concepts from Sampson and Laub’s [37,59] age-graded theory of informal social control are applicable to homicidal juveniles, a population of juvenile offenders that was not examined in their research on desistance from crime. Stable employment, positive intimate relationships, and human agency emerged as strong contributors to desistance, as evidenced by the direct statements made by the desisting offenders in their interviews. Both stable employment and involvement in a long-term intimate relationship provided the JHOs with a sense of purpose, which reduced their likelihood of engaging in behavior that would endanger their access to the source of that purpose. Additionally, prosocial intimate partners served as direct sources of guidance and supervision by encouraging the JHOs to avoid decisions that may lead to adverse outcomes (e.g., Lima and Golf).

The prevalence of human agency among the men in the sample who had not been involved in serious criminal behavior since their release from prison suggests that desistance from crime often involves a deliberate decision to change. The presence of turning points away from crime [59], such as a full-time job and a positive marriage or intimate relationship, may not lead to desistance if the offender does not consciously choose to desist and takes active steps toward achieving this goal.

The finding regarding generativity is consistent with several prior qualitative studies on desistance [42,44,60]. Similar to the findings reported in prior research, generativity facilitated desistance in the present sample by giving offenders a sense of purpose. Individuals are less likely to jeopardize their freedom if they sense that their life provides some type of benefit. Among the desisters in this sample, a sense of purpose was evident in two ways: (1) ensuring that juveniles refrain from engaging in criminal behavior and do not experience victimization in prison; or (2) working with prisoners to help them readjust to society after release.

As discussed in the literature review, identity transformation has been linked to desistance in multiple prior studies [38,39,41,42,44,61,62]. This concept involves the perception that one has control over his/her life and the development of prosocial values. The present study provided mixed evidence for the relevance of identity transformation in the desistance process. On one hand, the desisters sensed that they had control over their actions and destiny, as demonstrated by the statements presented in Section 4.1.4 above. On the other hand, the follow-up interviews provided little to no evidence of the adoption of prosocial values by the desisters. The lack of involvement in serious criminal behavior by these men appeared to stem less from a change in values, and much more from the desire to avoid future incarceration and exposure to positive external circumstances (e.g., stable employment, the absence of neighborhood-based negative peer influences, a positive intimate relationship).

### 5.1. Developmental Maturity and Desistance

Support has been found in this study for the effects of age and maturation on desistance. The persistent offenders in the sample, who were not examined in depth in the present study, served significantly less time than desisters (Chi Square (1) = 6.134, *p* = 0.13, Phi = 0.57). The mean time served by persistent offenders was approximately 96 months (8 years). This datum indicates that the persistent offenders, on average, were 25 years of age or younger at the time of release. Desisters, in contrast, served 247 months (20 years 8 months). A perusal of the length of time served by desister/persistent offender status is illuminating. Six of the eight (75%) desisters served sentences of at least 15 years, whereas nine of 11 (82%) persistent offenders served 8 years or less. Accordingly, desisters were considerably older than persistent offenders at the time of release; on average, they were at least 35 years old.

The difference in average time served strongly suggests that desisters were developmentally more mature than persistent offenders when they were released from prison. Accordingly, they were returned to society at an age in which their prefrontal cortex had likely already been fully developed and were in a better position to make more rational decisions and to control strong emotions [63]. It is important to note that, despite having served more time and being older, the desisters in the present study were at risk in the community longer than the persistent offenders. On average, the desisters were in the community approximately 10 times longer than the persistent offenders (213 months—12 years, 9 months—compared to 21 months). The finding that JHOs who served longer sentences were less likely to recidivate is consistent with other studies [8,9,30,31,32]. The influence of aging on desistance among JHOs should continue to be examined in future research.

### 5.2. Implications for Criminal Justice Policy

The results in the present study provide several preliminary implications for policies that would benefit formerly incarcerated homicide offenders and may be effective in reducing their likelihood of recidivism. For example, given the high prevalence of desisters who made a change in neighborhood after their release from prison, resources should be devoted to helping JHOs settle in a neighborhood other than the one in which they lived prior to incarceration. Similar to the findings in this study, research from other states (Louisiana and Maryland) showed that deliberate efforts to relocate released offenders away from their old neighborhood resulted in a lower likelihood of recidivism, compared to released offenders who settled in their old neighborhoods [64,65]. Moreover, the findings presented by Kirk [64] suggested that the farther offenders move away from their old neighborhoods, the less likely they are to recidivate. This relocation policy should be expanded to help offenders in avoiding the adverse influences (e.g., antisocial friends, a high prevalence of illegal drugs, previous reputation) in their neighborhoods that contributed to the original homicide offense.

The influence of stable employment on desistance demonstrates the importance of reentry programs that teach job skills to young violent offenders and provide other employment-related services, such as guiding offenders in their search for a job. Essential job skills include appropriate behavior during the initial interview, non-aggressive methods of communication with a supervisor and other co-workers, and effective time management. One such nationwide program for violent offenders, which is referred to as the “Serious and Violent Offenders Reentry Initiative”, has produced promising results with respect to its ability to reduce the likelihood of recidivism [66].

Exposure to a reentry program during incarceration and after release can contribute to desistance among JHOs, and young violent offenders in general, in multiple other ways. This type of program can increase an offender’s frustration tolerance and improve his/her communication skills, both of which may facilitate the development of an intimate relationship with a prosocial partner. Moreover, if the program includes rehabilitative treatment such as cognitive behavioral therapy (CBT), it may lead to desistance through changing the offender’s belief system [67], and consequently encouraging him/her to take conscious steps toward a prosocial lifestyle.

### 5.3. Limitations and Directions for Future Research

The present study consisted of a unique investigation of the changes in the lives of men who committed a homicide offense as adolescents, and it provided a preliminary indication of the important factors that contribute to desistance among JHOs up to middle adulthood. Nevertheless, there are several limitations to this study. The primary limitations are the small sample size and the fact that all the JHOs in the sample were from the same state, which limited the generalizability of the findings to young male homicide offenders in general. A qualitative study with a larger sample of JHOs released from prison would better illustrate the ability of the factors identified in the present sample (e.g., avoiding pre-incarceration neighborhood, stable employment, human agency) in producing desistance among formerly incarcerated homicide offenders in general.

Moreover, homicide offenders, and convicted felons more generally, do not face the same circumstances in every U.S. state after they are released from incarceration; for example, barriers to employment for felons vary from state to state [68]. Therefore, future studies should conduct long-term examinations of JHOs from various states, which will clarify whether post-incarceration outcomes substantially differ based on the state in which an offender is released.

The generalizability of the results may have also been impacted by the fact that the JHOs in the present study were all released from adult prisons. Confinement in juvenile correctional facilities, which prioritize treatment and rehabilitation to a greater extent [69], may have increased the amount of desisters in the sample and altered the factors that most influenced desistance. Future research would benefit from examining the differences in desistance between JHOs released from adult prisons and those who are released from juvenile facilities.

Lastly, the offenders in the sample were all men. There is little to no knowledge currently regarding female JHOs’ post-incarceration experiences over the life course. Accordingly, the post-release lives of girls who commit homicide offenses should be analyzed in depth in future studies, to assess whether similar factors (e.g., settling in a new neighborhood after release, the avoidance of pre-incarceration peers, stable employment, positive intimate relationship) lead to desistance for this group of offenders as well.

## 6. Conclusions

The analyses presented in this study highlight the importance of both external and internal factors in the desistance process of male JHOs. On one hand, it is important to isolate released JHOs from the antisocial environment that led to their criminal behavior during adolescence, and expose them to prosocial peers, intimate partners, and employers. On the other hand, desistance may not take place if the offender does not decide to change his life and stop engaging in behavior that carries the risk of further incarceration.

As discussed by Heide [32], all the offenders in this sample viewed prison as a violent and threatening place. The particular circumstances that a male homicide offender needs to experience in prison before deciding that future incarceration is not an acceptable outcome have not been clarified to date. Future research on homicide offender recidivism would benefit from exploring this issue in depth.

## Figures and Tables

**Table 1:** Demographic characteristics and homicide conviction type (n = 19).

Variables	N (%)	M (SD)
Offender Race		
White	6 (31.6)	
Black	13 (68.4)	
Offender Age		52.47 (1.17)
Age at Homicide Arrest		15.68 (0.82)
High-Crime Neighborhood		
Yes	8 (42.1)	
No	11 (57.9)	
Homicide Conviction Type		
Murder 1	2 (10.5)	
Murder 2	12 (63.2)	
Manslaughter	1 (5.3)	
Attempted Murder	4 (21.2)	

**Table 2:** Childhood maltreatment and prior record characteristics (n = 19).

Variables	N (%)
Evidence of Childhood Abuse	
Yes	9 (47.4)
No	10 (52.6)
Evidence of Childhood Neglect	
Yes	14 (73.7)
No	5 (26.3)
Pre-Homicide Criminal Record	
Yes	15 (78.9)
No	4 (21.1)
Pre-Homicide Violent Record	
Yes	8 (42.1)
No	11 (57.9)

**Table 3:** Homicide incident characteristics (n = 19).

Variables	N (%)
Use of Accomplices	
Yes	15 (78.9)
No	4 (21.1)
Stranger Victim	
Yes	11 (57.9)
No	8 (42.1)
Homicide Circumstances	
Crime-Oriented	14 (73.7)
Conflict-Oriented	5 (26.3)
Weapon Choice	
Firearm	8 (42.1)
Blunt Object	4 (21.1)
Knife	4 (21.1)
Strangulation or Asphyxiation	2 (10.5)
Multiple Types	1 (5.3)

**Table 4:** Factors in desistance for each JHO (n = 8).

JHO Name	Avoiding Old Neighborhood	Avoiding Old Friends	Positive Intimate Relationship	Stable Employment	Generativity	Human Agency	Total
Golf	X	X	X	X		X	5
Echo	X	X			X		3
Lima		X	X	X		X	4
Romeo	X	X			X		3
Mike	X	X	X	X		X	5
Hotel		X		X			2
Victor	X			X		X	3
Oscar	X	X		X	X		4
Total	6	7	3	6	3	4	29

X = factor is applicable to JHO.

## Data Availability

Study participants are all convicted felons who agreed to participate in this study provided that their responses remained confidential. Accordingly, the data used in these analyses are not publicly available. Pseudonyms are used throughout this manuscript. Interested parties may contact Heide with questions about the data.
